# Lectures on the Duties and Proprieties of the Dental Practitioner

**Published:** 1855-10

**Authors:** E. Townsend

**Affiliations:** Philadelphia


### The Dental Register

### OF THE WEST.

VOL. IX. OCTOBER, 1855. NO. 1.

### Original Lectures, Essays and Communications.

### LECTURES ON THE DUTIES AND PROPRIETIES OF THE DENTAL PRACTITIONER.

By Prof. E. Townsend, D. D. S. Philadelphia.

At the meeting of the American Society of Dental Surgeons held in Cincinnati 8th and 9th of May last. Dr. E. Townsend by special request, delivered two Lectures which he had prepared for the Dental Class in the Philadelphia College of Dental Surgery, on the duties, proprieties, courtesies &c. &c. of the Dental Practitioner.

Many members of the Mississippi Valley Association also then present, joined in a request with the members of the American Society, that the lectures be published in the Dental Register. In accordance with this request, Dr. Townsend has kindly furnished us a copy, and we feel that the intrinsic excellence of these lectures on a subject so important, is more than sufficient apology for occupying so much space with one article in the present number of the Register. These proprieties of professional life are important not only as necessary in consulting the feelings of our patients, but a neglect of them gradually blunts our better sensibilities and renders us incapable of appreciating those feelings which are the result of a correct taste and fine education.

The man who is slovenly and dirty in his person and loads his breath daily with the fumes of Alcohol and Tobacco, will soon be unable to appreciate the finer sensibilities of the cleanly, who cannot but wish to avoid a too close proximity to that which is so offensive.

To the young practitioner, whose aim is *success*, these lectures will be of incalculable service. They come from one who has been long enough in the profession to realize and profit by the adoption of that which he recommends.

An uncouth manner, a dirty hand and napkin, and a foul breath has impoverished many whose scientific qualifications might have insured success.

Gentlemen:

The chair I have the honor to occupy, and endeavor, as I best can, to fill, owes to the class which honors its officer with their respectful attention, both duty and respect.

These obligations belong to our professional relations, independently of those personal regards which arise out of the very pleasurable intercourse necessarily subsisting between us.

This intercourse and intimacy, now near the close of our first session so well established, I am happy to acknowledge for myself, and to believe for you, have strengthened and deepened the feelings which the rules of etiquette between teacher and pupils, are designed to recognize and maintain.

Especially happy am I in the assurance which I feel, that every proper discharge of my duties will receive in your minds the kindest construction its faithful frankness may perchance demand.

I have no right, and I shall never presume upon my privileges of position to speak to you upon any topic, nor with any purpose, nor in any manner from this place, other than belongs legitimately to the chair which I hold.

One branch of this, my duty, cannot be divested of such personality as attaches to the application of the abstract principles of professional conduct to the practice of those who are about to put themselves within the range of their requisitions.

To the chair of Practice of Surgery, and of Obstetrics, in the medical colleges, belongs of right the duty of suggesting the proprieties of professional conduct and demeanor in the sick room, and in the general personal relations of the practitioner to his patient. The teachers of these departments speak without hestitation to their pupils upon this important topic, and I have the warrant of their example, as well as of the reason and justice of the thing, for the similar duty of calling your attention now to the equally important matter of the Dentist's personal relations to his patients, the decorum required and the influences exerted both upon himself and upon them.

I do not suppose that rules covering all the points involved can be definitely laid down, nor that I am capable of delivering the oracles of a perfect demeanor, in all the circumstances deserving to be considered and provided for, but I will not decline the task because of its difficulty, or leave unattempted a duty so important on accouut of its delicacy. That which may be clear and just will commend itself to your good sense, that which may be lacking you will endeavor to supply, and the decision of your own judgment and taste must be your protection from the mistakes of mine. I assure myself, that however this difficult and delicate duty may be performed, good must come of the hints and helps which shall put us all upon inquiry in matters of such importance as these. Do me the justice, however, I beseech you to receive my thoughts as wholly impersonal in my intentions, and, do yourselves the justice to make them personal, so far as they are worthy and can apply; but, all at your own risk and responsibility. You are entitled to my opinions, to the results of my experience and reflections, and, you are also entitled to your own proper liberties in judging and accepting them. I speak *to* my subject, not *at* my audience, and you must think only of what I say, and what that may suggest for your own guidance and government, and not of the speaker as of a personal acquaintance. A generous confidence will give me my proper freedom, and will afford you whatever benefit can result from its frankest exercise.

Although I have used the precaution, before entering upon this delicate and debateable ground, of taking more care of the forms of expression than extemporaneous composition secures, I freely confess, I have not been able to construct a code, or arrange an essay, with the fullness and accuracy which the subject demands. But the impulse and the obligation are alike upon me, and I must do what I can, as well as I can, caring more for the substance and effect of my remarks, than for their systematic symmetry.

Added to all the intrinsic embarrassments of my undertaking I am obliged to find my way through it without the assistance of any guide which I can make available in the route to be pursued, and I proceed therefore with the caution and doubtless much of the irregularity of one who must *explore* a wilderness while he pursues his journey. Enough of hestitation. explanation and apology. Let us turn to the subject, or subjects which we are to consider.

First of the office: ante-room, furniture, reception of visitors, and their accommodation and entertainment, generally and particularly; or as the Lawyers say: "of property and appurtenances, real, personal and mixed.

The general character, the location and style of your residence must of course be governed, at least limited by your means, when *they* are limited; if funds or credit abound, substantial elegance governed by good taste cannot well run too high. The gentleman and his banker must settle these outlines, and make the best compromises, which any particular case requires.

Economy is a virtue and duty as well as necessity, but style is a good investment in our business, and an important and productive part of the stock in trade, much of our work is a matter of appearance to our patients, and our establishments should be in good keeping with the sentiment which they are concerned with. The properties and appointments of art ought to be artistic and attractive. These harmonies are more felt and noted than inattentive people are apt to imagine. The office or operating room, is more within control and its proprieties more nicely exacted, than those of the general edifice.

It must be as little like a workshop as possible, and it must not in any degree be converted into a lumber room. It is not the place for a convention of horsewhips, old boots, shotguns, pamphlets, fragments of the last lunch, and ingenious rat traps in progress of construction; nor must it look terrible, for the sake of looking imposing in its array of instruments of torture. Display in *itself* is a vulgarity; in a Surgeon or a Dentist it is barbarous.

The bee hides his sting till he must use it; and the snapping turtle who uses *his* forceps rigorously enough upon occasion, knows how to shut up his case, and they are both all the more beautiful and amiable for such consideration. Monstrous three or four fanged molars handsomely extracted, are trophies of skill to be sure, but they are a regular night mare to the unhappy wight, who may have one aching in his jaw. Keep your tools out of view, a well constructed case will enable you to do this with a turn of your hand, when you are not actually using them, and place it so, that your person will hide most, if not all of it from your sitter in the chair; and let no sign of bones and blood oppress the sight or shake the nerves and offend the feelings of your patient. A patient of mine within a few weeks left an expert operator, because she saw blood on a pair of forceps, which she believed had been thus coated when used in her own mouth. She could endure any reasonable amount of pain like a soldier, but she did not relish the sight or taste of other people's blood. Your office should have the air and arrangements of a parlor, or of a boudoir as nearly as possible, and be kept scrupulously free of the associations arising out of its daily use, and offensive service. Its furniture should be rich, neat, simple, and free from an encumbring quantity; two chairs at farthest, with an ottoman, and a centre table for books for the entertainment of company. One wash-stand for your own use, but no jumble of tooth brushes of your own, or other utensils for lazy convenience, when patients are absent. The office must be dedicated to operations, and guarded against all other uses. It is no place for pictures of yourself with a set of teeth in your fingers, or mantel piece arrangement of artificial teeth, grinning like dissected jaws at those who are only too conscious that their own are to be cut and carved after a pattern. One fine bust of *ideal* beauty, or one superior picture in the line of the sitter's gaze when in the chair, may be effective and proper, but be sure that it *is* a fine one. Of all things be sure not to show bad taste in form and style of your ornaments.

The like principles of taste and propriety should govern the order and arrangements of your ante-room. In summer it should be supplied with fans, which should be neither mean nor absurd; nothing flash, and nothing beggarly about them. The books on the stand and center-table should be of standard authors. Not exactly those that are on every table in town. The last history of the world, and "the rest of mankind," the newest novel, nor the most elegant trumpery of the last holidays. It is not the place for the city directory, nor a patent medicine almanac.

There are enough of works of *classic* authors, whose names every body knows, but few read, except those who pilfer from them for the best things in their own original productions. Lay a few of these within reach of persons who fill up all their time with wet newspapers, and uncut cheap editions of the last agonies of literature, and they will *remember* the minutes they spent in waiting, and connect you with a rich enjoyment and a good impulse ever afterwards. Dont let book-hucksters dictate your reading, and help your friends to a hint of better things. There should be no Dentistry or Surgery in this ante-room, either in books, pictures or crockery. There ought to be no particular, especially no peculiar, or partizan drift in the provision of books. If you *have* a hobby, never solicit any body who comes to you professionally, to ride it; bore your friends, for, they bore you in turn, but let other people alone; newspapers, tracts, memorials, petitions, and all sorts of contribution boxes are to be banished from this chamber, and with them all the impertinence of sects and isms of the day. Your patients suffer enough in their teeth, without having these things thrust into them, or along with them. Provide works which will serve to pass the time of waiting for almost every class of persons or of mind that may need such pastime. Let them be rather above than below the level of mediocrity. Let it be your habit to *level* up in all things, not down: You will thereby preserve and mend your own tone of life and thoughts, and impart while you impress it upon others. The effect will pay every way. Out of such things arise those regards and respect which dignify life, and give personal character, command of criticism, and authority for all useful exigencies. The idea that runs through these suggestions is, that you are to be a gentleman as well as a dentist, and you are to fortify yourself in that position by all your surroundings, and deepen its impression by every condition of your professional life.

Servants should be trained to receive visitors with politeness; and the politeness of such service is not mannerism and flourishes, but promptness, alacrity, and precision, with earnest respectfulness. A servant should not be servile, that spirit is too mean and cheap to be agreeable to others or creditable to yourself. A respectable man is indicated by respectable as well as respectful domestics. It is a fact that they always reflect the manners of their employers, or indicate them to a quick eye by the demeanor which is exacted of them. They behave to people at the door as he would do, or else they show the falseness which he requires of them for his own purposes. Such unveracity is concealed from no body that is *worth* deceiving. It takes an artful man to play his own tricks successfully, *he* always fails in the loug run; and servants are *sure* to bungle and expose them. Let no man ever take such assistants into any of the little frauds and conspiracies of habitual evasions and hypocrisy.

Have a bell in your office which a servant can touch in case of a call while you are engaged, to prevent knocking at the door, and the delivery of messages and *names* in the presence of your sitter. Besides the importance of avoiding disturbance, at perhaps a critical point in an operation, it will answer another excellent use. Patients are sometimes very *im*patient, and persons having business like to be despatched, because they came, not to wait an appointed hour, or to take their turn when an interval occurs. Such persons after waiting a few minutes will ring again for the servant and request you to be re-informed of their presence. Let the servant repeat the signal, or assure the party that you will answer without any avoidable delay, or let the one intimation be the rule, and the servant retire without either promising or refusing to repeat it. You must do justice to all parties. The sitter has his claims, and they are first in order. You are the best judge of rights and proprieties, and you will never lose anything by doing just right. If you observe your own rules, your household will do so also, and all your sitters will learn to respect them, and to conform for the sake of their own interest in them. You will save your own temper too, for men are seldom impatient of things that they know how to defend themselves against. A little breeze does not disturb a sailor, if his ship is in trim,—all taut, the ropes in order, and the helm in his own hand. Dont break your own rules, and then fret because they are broken. Nor are you to be the slave of rules, either. If they are well made, they need not be so stiff as to lose all accommodation to exigencies. The magnetic needle fluctuates under disturbances, but is true, all the while, to its polarity, it always settles to the right point of the compass, and the sailing is as safe as if it were in a bee line all the time.

Convenient accommodations should be provided in the hall, if possible, for coats, umbrellas, overshoes, &c., for gentlemen, and for ladies in a room contiguous to the office. Never furnish any place with comb and hair brush. Dont allow any body to believe that you think such a practice cleanly. You cannot give a new one to every visitor, and you might almost as well offer the loan of a tooth brush, or do as I have known to be done—scour their teeth after other operations were completed, with a brush which had done the same service in many another mouth before. I limited the chairs in the operating room to two, because the presence of a single friend *is*, sometimes, admissable, or even desirable. *One* witness of an operation is enough, more are troublesome to the operator, unless he can effectively play off one against another.

But we seldom find people who can keep their hands to themselves, and as seldom those who can use their tongues without injury to our patients, and our own management of them. I dont say that a dozen of the right kind of persons would be too many anywhere, but I dont suppose *half* a dozen will ever meet in any operating room. The effect of witnesses upon patients varies very much. Sometimes you will find it best to request your patients may come to you alone, to have the best success. This for various reasons. Some people, mothers particularly, are very injudicious in their sympathy, and often behave in such a way as to make the child feel as if he or she were enduring something very horrible, with worse to come; and therefore may well be excused for some fuss. Others, with as bad policy, and as little judgment, will not allow any expression of distress and fear, or any shrinking from pain, for fear of a total failure of the operation. They either cannot judge, or do not consider the truth and right of the case, and so their influence, unjustified by the child's own feelings, is good for nothing and worse.

Either of these extremes is bad for the patient, and the dentist, if he is skilled in the management of his subjects, patient in his own spirit, and properly considerate of the circumstances of the case, will know how to adjust himself to it, and will do it most successfully, in the absence of incapable friends. He will know how to be gentle and sympathizing where it is due and expedient, how to be rigid and determined where such a demonstration will supply the lack of courage, and steady the patient's resolution. His practice should make him familiar with the ropes, and undisturbed he ought never to fail, and seldom will. His little sitters alone with him, relieved from the presence and influence of those, who mayhap, have done him a thousand other injustices by their mistakes, whose weaknesses and faults he knows and counts upon better than they think, will, in the hands of a judicious practitioner, whom he supposes, knows every thing, and is just and kind, be like a puppet to be played with at will, for their own best advantage. Indeed, the operator may give children such lessons in conduct, and such an experience of self government, as will render them yeoman's service for the rest of their lives, in matters of a different kind, and of higher moment. It is often in the least considerate events of a young life, that the impulse and direction of its after history is to be found. Think of these things as well as of your strictly professional duties and interests.

Conversation is about as difficult as dentistry itself. Teeth and tongues are not very easy of the best management. The sitter's tongue you have your own way of holding for him. Over his friend's you have less control, and then, excuse me, there is your own to manage, and I have something to say about that.

It is safe to say, that all conversation during your work and its little intervals, should be upon general topics, as far from plugs and nerves—particularly the nerves, from files, forceps, and flash operations as possible. *They* are near enough in *fact*, without being intensified with *talk*. The theme should never be upon the operator's own manner of performing his manipulations, and his superiority over all others in the known world. No doubt it is all very wonderful, but then every body knows you think so, and you can spare the brag and open egotism of it, without any danger of being charged with undervaluing yourself; and what is far better, you will avoid that most unhandsome, and most foolish practice of undervaluing other men in the profession. It is best as a rule never to lecture either gossip or professor fashion to your patients about their cases, or anything else in your own art, unless they desire information which it is proper for you to give them. The accomplished dentist should so store his mind with the general knowledge belonging to a gentleman and man of science that he shall not need to be, on all occasions, in season and out of season, talking of his calling.

When direct inquiry is made, let him remember that it is practical information the patient needs, not a Fourth of July oration, or a flourish of words and scientific terms and phrases, to display his erudition. Ardor and zeal in the study and improvement of one's profession is apt to lead him beyond the boundaries of good taste in talking about it. Guard yourselves well there,—and especially remark, the use of technical words discovers rather your estimate of them and their supposed effect upon others, than your own deep and familiar acquaintance with their significance and proper use. Well grounded and habituated thought is not so fresh and green, or so new fangled as recent and imperfect discoveries show themselves. Remember that all pretence is vulgar. Avoid the very appearance of it.

As a general rule for your demeanor and style of address and conversation to your patients, especially to men, professional and unprofessional, distinguished and unknown, gentle and simple, bear in mind that science and art, like religion, is no respecter of persons. A physician, surgeon, or a literary savan in the chair *is* your patient and nothing *more;* a gratis patient or a servant is your patient and nothing *less.*

Such accommodation as adapts your bearing to all varieties of men is of course required in all kinds of communication, but take care of anything beyond this, anything with a different object and aim. One of the worst mistakes a dentist can make is to talk flash technicalities to a medical man. If he is not a snob, he wants no such display, and if he is, he wishes to display himself, and you will make nothing in such a contest. If your sitter is an ignorant person, or what is the same thing, ignorant of anatomy and physiology, he already credits you with knowing your profession, and, if he is a man of general culture, he has a quick sense of the proper bearing of a cultivated mind and its self-balanced dignity. He has seen enough of *astonishing* people, and enough of egotism, and he knows their measure and meaning. As you are not to flatter a distinguished sitter, so you are not to fear him. Is he a first class surgeon? No matter. He may not have a tythe of the skill in his art that you have in yours, and if he has, he of *all* men best knows the deference *due* to every man in his own calling. There he is, a man simply, with the toothache, unable to help himself. There you are, a dentist to treat his case. Take your own position and keep it. When he asks you for a reason, or a prognosis, give it to him, respectfully, modestly, but in the fashion that asserts your own right to settle the matter. If *he* has an opinion also, hear it of course, but act upon it as he would and should upon yours when you are his patient. I do not say that you should not be helped to a judgment of the case from him, and I do not say that you should not learn even from his coachman, if either has anything to suggest, as they both very well may have. But recollect your position.

Consultations with surgeons and physicians upon cases under their or your treatment, are a different matter. They come to you for your observation and experience inthose diseases which involve the teeth.

Here you are to be candid, guarded and unaffected. Be confident where you are clear, be sure where you can convince, be firm where you are satisfied. Do not consent to perform an operation which your own judgment absolutely condemns. A surgeon would not touch a limb with his knife if he believed it ought not to be amputated. Why should you act as the mere instrument of a surgeon's ignorance. Assert the equal rights of your own profession within your own sphere.

You have the conscience of a man, and the dignity of a profession to maintain. We wish our diploma to stand for what it claims in the conduct of your professional life. Dentistry is surgery, and if it were only stage driving it has a right to the track, and should "keep to the right as the law directs." In such consultations you give your opinion if it is asked, and you will carry it out in practice if required; or you will ingenuously and frankly modify such opinion for sufficient reason. You will also act as assistant, merely, in some great operation, just as a surgeon will perform an operation for an accoucheur, who lacks the skill without taking the responsibility of the entire case. But when the whole range and issue of the case is entirely within your province, the proper obligations and the independence of your science demands that you shall not sink your profession into a blind and discreditable submission. In all such consultations, whether the case is yours or the surgeon's, keep within your own department, even when your general medical and surgical knowledge is the superior of the two. It is dentistry that you are representing,—dentistry only, with all which it includes, and it is but just, modest, and safe to keep within the line. Even success beyond your own borders of duty will be regarded as a sort of impertinence, as much so at least as the interference of the surgeon with your own proper department; and a failure is fatal to an intrusive audacity. The principle which lies at the bottom of this rule of conduct is, that all assumptions in callings not strictly your own, are of the nature of quackery, as much so in regard to other branches of the healing art, as in law, theology, or military science. You should know all the branches of general medicine, but you must practice only your own, *its* responsibilities you must take, and the necessity will justify or excuse even your mistakes there, but nothing warrants usurpation of other offices and duties. The best procedure is not to claim any thing beyond your proper range, and if you are, nevertheless, qualified it will be perceived by the discerning among those whom you meet, and they will credit you with it, and with the delicacy besides, which duly respects their rights and honors.

Young practitioners are very apt to defer too much to persons of rank and pretension, in their chairs, and this may arise from feeling the importance of pleasing. and so securing their patronage. Even when modesty dictates this manner, it is an injurious weakness. When it is policy, it is, to say the least of it, a sad mistake. In justice to your setter, you must not allow yourself to be swayed in the matters where you are the most competent to think and decide. And it is just as true that no man, however he may relish subserviency and flattery, wants such weakness in the man on whose knowledge and skill he feels his own dependence.

I can add but one suggestion now upon this theme, so ample, and so important that its thorough exposition deserves a treatise instead of a mere passing notice,—and that is—be ever more intent upon your work, than upon your fame. Do your *duty* well, and that will take care of your reputation and your prosperity at the same time. You put your patient into the position of a mere subject of your art, whatever he may be, carry out the idea while he is in your hands by thinking of *yourself* only as the *operator* in the case. Your praise becomes any other man's mouth better than your own, and if *your* work becomes *their* mouths, their buccinators will blow your trumpet with deserved, acceptable, and genuine commendation.

Your *operating chair* should be so constructed that the patient shall fill it, that is, not so wide as to allow him to slide to the farther side of it, with an inconvenient distance between you. In such case you are obliged to twist and overreach so as to fatigue yourself before half the day's work is done, and much disagreeable awkwardness of position, and many a bad and imperfect touch and pressure results from it. But I mention this now especially for another purpose. The chair should permit the closest proximity of your person without the risk of accidental contact. A majority of our patients are ladies, and although they may submit, it is impossible to say how disagreeable personal contact may be to them. Disagreeable or otherwise, avoid it scrupulously. No matter what *your* feelings are, recollect that it is the most sensitive who are to give the rule. The finest ear, the most delicate smell, by right of superior fitness, prescribe the law in music and perfumery, and the duller and more indifferent sense is bound to accept it from them. It is not every man who understands and feels, the sweet, chaste, sacredness of the person; and offences against it are everywhere but too common. In our profession more than in any other, discernment and delicacy are most invaluable and most obligatory. We are constantly occupied with our patients, in such way as to bring us into a sort of nearness which is easily overdone. Even the gentleness and soothing kindness of manner which we must often assume, readily passes into over-tenderness and familiarity. Our manner toward timid patients may become too caressing. When this over-sweetness and nearness is only a make-believe, it is contemptible to a lady of discernment, when it is a little more real it is simply an outrage: and let no man flatter himself that he is wholly secure from detection in any impropriety. Mere coarseness and bluntness of nerve is repugnant and offensive; indifference is insolent, and presumption is insulting. Nay, a mere doubt as to which or what it is, disturbs and distresses more than a lady's self-respect ean well endure, though it will not acknowledge or intimate it. Here again the general rule comes in for our security when we have no more specific guide. Your patient is your subject, an impersonal object upon which you are employed as a work of art, and nothing else. Let me hint a particular or two for the general suggestions they afford. An accident disarranges her dress, and she will herself detect it, perhaps with a little mortification, so soon as she is relieved in her posture, or her nervous disturbance is quieted. Restore it if you should for her relief, but do it with no feeling of impropriety, and with nothing of the manner which indicates such feeling. Dont subject her to the distress of discovering an exposure which she should avoid, nor rectify it as if it were an awkward affair.

Take another incident which may possibly happen. A piece of your gold, which you may need to use, slips from your forceps and falls upon some exposed part of her person, which ought not to be touched. If you *must* have that piece to finish your work, and injury would result by letting the mouth rest until you prepared more, then lift it, as you would from your own table, or any other *insensible* surface. Let the necessity justify the act, let the manner conform to that necessity, and there is no indelicacy in it, and none will be perceived or felt. Just *be* right, and you will *do* right. Guard yourselves from accidents and their awkwardness by the highest rules, and unavoidable things will bring their excuses with them. One thing more, your patient however insensible or indifferent to decorum, is still only your patient. Treat no one in your chair with the soft nothings which do not become it. Carry the best habit of professional conduct into all your professional work, and you will have the benefit and happiness of it in many an unexpected way.

Believe me, gentlemen, I speak only from convictions of duty, credit me with no little opportunity for judging, and no slight impressions of the importance of these hints; and allow me to say also; that as the ten commandments, though they forbid lying and stealing, covetousness and murder, are no insult even to those who do not need such prohibitions, so the ideas of decency and duty which I submit, are in no proper sense an impeachment of any gentleman's taste, delicacy or integrity. They are things deserving to be known and considered, and therefore not improper to be uttered. I know my own motives, and you will not misjudge them.

Feeling the danger rather of overlooking matters worthy of note, than of overloading the discussion with particulars, before I dismiss our special relations and conduct to our female patients, let me suggest another matter which ought to be considered: Ladies have their own motives and perhaps feel some necessities which govern their intercourse with each other that we neither know nor can understand, if the ladies of your own family must of necessity meet your patients in hall or parlor, it should be understood, that patients are not visiters, and that no necessary politenesses imply acqnaintance and familiarity. They should be involved in nothing that they do not themselves contract for and distinctly invite.

In making your appointments, and receiving and dismissing them, have an eye to preventing all avoidable encounters of ladies who do not know each other or do not wish to meet, or to be met at your office; never *compel* any intercourse even between sisters which you can avoid, you know nothing of the relations, the little troubles and embarrasments that may exist among them; make every one feel as safe as may be from anything and everything but that which she comes to you for, and rest assured that such consideration will pay —in just apprecaition and in business prosperity. The simple right of the matter is, that every lady is entitled to her exclusiveness, for it may be of the highest moment to her feelings and her plans, and her dentest should not be a catastrophe to her.

*Add* to all this, that out of your office you have no business to recollect that she ever was in it, that she has a bad tooth in her head or that you made the handsomest ones she wears.

Now there is nothing in all this that she can or would ask you to observe, but not a point in it which you would not be censured for neglecting, and that, as far as you might avoid it, deservedly.

The Queen of Sheba after a right royal confab with king Solomon, cried out in astonishment at the wisdom of the great professor of "proverbial philosophy," that "the half had not ibeen told her." In quite another sense of these words that is your situation, exactly, gentlemen, I mean that you have not heard the half that I have to say on this general subject, but whether your astonishment is like hers or of a very different kind, it must be continued. I have more of the same sort in preparation for you, and when we meet again will proceed just as if both parties were equally well pleased with the entertainment.

---

### 2d *Lecture—Generalities of Professional Conduct—Order of the Office, &c.,*

We are not yet quite done with the office, its properties, and appointments. The attentions due to the ladies, very naturally led us a little aside from the main drift, as it would be laid down in a regular inventory of particulars, and we will now return to it for such observations as remain to be made, and I have time to present to you: First, some minor matters which I will not overlook, because I would not have you by any chance neglect them. The spittoon of the operating room should be as capable as possible of being always nice. It can be so constructed as to remove with the greatest celerity all blood and other offensiveness from the sight of the patient, and relieve yourself from all disagreeableness of that kind which usually attends our operations. The patients, perhaps, feel more for such exposure in the presence of a gentlemanly operator than on their own account—do your best to relieve them in this matter.

The fewer instruments laid out for use the better. They should be returned to their respective places every night, I mean perfectly cleaned, and put into good order, taking them out with your own hands every morning, will enable you better to recollect through the day exactly what you have on your table, and you will be spared embarrassing searches for those which you will need. If you pass one by one through your hands before you begin your work, they will be at hand in every emergency. Besides, if they lie on the table from day to day, they will accumulate dust, fibres of cotton, and other small trash. Lying upon each other as they are dropped from the hand for irregularly long periods, they stain and defile each other, and the fault is concealed till the moment that they are to be used, and besides, with such negligence and disarray, they get altogether an untidy look that is unbecoming and unpromising to the eyes of your patients. The same untidiness is evinced by a want or scarcity of clean towels and napkins. These should be abundant, and of the best and whitest linen, carefully washed and folded, and always at hand ready for instant use; my own stock reaches full forty dozens, and I would not be contented nor comfortable with a much smaller number. It is a common remark by our lady patients, that they would prefer such or such a dentist to operate for them, even if he had less reputation for skill, because his napkins are so clean, sweet and fresh, that they are sure it is a principle with him in everything. *This* qualification any practitioner may easily command, whether in everything else he is quite superior or not, and as it atones for some want of skill, *have* at least that which it is inexcusable to want.

This brings me naturally to the person of the operator, under protest as before, that it is not your speaker, but the *subject* that is personal, with the application all afloat, till the proper person for himself appropriates it.

The dress of the dentist should always be clean and of a kind that *looks* the character. That cleanliness should always be fresh too. The coat should be of a material that will wash, and of a color that would *show* dirt and not suspiciously conceal it. The *fact* with its best *evidence*, for the better assurance of the party concerned. The hair of our sitters is often greased; the arm necessarily rests upon the head, and the sleeve then contracts a soil and smell that is very offensive to the face of the next patient. If you wear such a dress as will expose the fault, you will always see and correct it. A woollen sleeve is a regular sink for such unrecognized filth, and the *frowzy* smell and touch are sure to apprise the patient of the offense. His hair if long and straight, should be so cut as not to fall like dipped candles over his own face, or that of his sitter; and that part of his face which he shaves should be cleanly shaved every day. His own mouth should be in perfect order, at least as far as concerns the perfect purity of his breath, and generally it should look so well as to answer for a promising pattern card in his own face. "Physician heal thyself," has a direct application to him. If it hits him slap in the teeth as a reproach, it will go far to "condemn him out of his own mouth."

The attitude of the operator ought to be that of a man who stands up to his work as if it were fairly before him; not that of a man who has a tussle with it, or is engaged at something which he never did before, and therefore knows not how to attack gracefully and with advantage. A surgeon who should thrust his right foot forward, to amputate a limb; with the knife in his right hand would show such embarrassment, or ignorance, as would convict him of unpardonable awkwardness. His thigh would be in the way of his elbow, his elbow would hitch on the thigh, and his head would be all wrong to his body, and that would be cramped and strained out of all freedom and self-possession. Wherever there is much twisting, there is too much friction, and too little liberty of action.

Many persons acquire a very bad and ugly habit of contorting the mouth and face, when they are doing difficult things. You will sometimes see it in persons writing, drawing, and playing upon musical instruments; but you never see it in the best performers in these several arts. It is a sign and confession of unskilfulness, as

Hamlet says to the players, "I pray *you* avoid it." It is generally accompanied with a porpoise like breathing, snoring, and blowing, from which the patient turns as soon as he can, while the unconscious operator imagines it is a gesture indicating pain, or a movement to relieve the fatigue of a constrained position. Train your eyes and face into propriety of behavior, and manage your chest, so that it shall not pant and wheeze like an asthmatic bellows; your face should be no more a tell-tale, to betray your anxiety and exhaustion than that of an accomplished gambler, who stakes his last cent without betraying the danger and despair of the hazard. This fault is chiefly owing to the habit of breathing through the mouth and between the teeth instead of through the nostrils. I care not how sweet and clear a man's mouth may be, it is an ugly habit to breathe through it upon the patient; and another very great fault, is to speak to your sitter with your face so close to his, that he must receive the current direct and feel it, and bear it, without chance of escape, except by apprising you of its unpleasantness, which he can hardly do. Don't clean your nails with your knife in the presence of the patient, and don't *wash* your hands, or brush your teeth there either. Do this in your private room always, washing hands and cleaning nails, because they are dirty, don't satisfy any body that they are clean, *there* is the dirty water to disprove it. *Lave* your hands in clean pure water in his presence, that he may see it is *done*, as a lady pours hot water from an urn into a tea cup at the table for further assurance of the guests. Among the habits which are offensive, are hawking, and then spitting to get rid of the phlegm—clearing the throat, nose blowing and the like; they need but to be named, and yet they do need to be named. I have only to say of them,

> "Oh, wad some power the giftie gie us
> To see ourselves as others see us,
> It wad frae mony a blunder free us."
> And ugly motion.

Here too, I may properly say to you, that all hurry, flutter, and bustle, under pressure of your engagements, are inconsistent with the best discharge of *present* duty. The case in hand is the *only* one that concerns the sitter, and in that *he* is too deeply concerned to accept a disturbed attention, or allow a distracting solicitude for the timely performance of other work. Let all your work be done with the properly assuring air of carefulness and attention. It is even fine practice in self-government to postpone anxieties until their time; make *them* keep their appointments also, it will be good for your patience, and it is due to your patients; manage your fretting, as well-bred women manage their crying or fainting, postpone it till you can do it up right.

An affected indifference, or a worrying slowness is as bad on the other hand. A natural earnestness, no more, no less, is the proper air, and gives the suitable movement to the method of the skillful operator. It tranquilizes the patient, it secures his confidence, and gives you the government of his nerves, and of his opinions too; and this last is a large part of your stock in trade. Depend upon it, men pass, very much as bank notes do, for what they promise upon their face, and their genuineness is ascertained very much in the same way, that is by the style, expression, and excellence of the engraving. I do not speak of these things as mere mannerisms or tricks—*good* faith and good conscience forbid; but as of *true* symptoms of *true* things—" the outward and visible signs of inward and spiritual graces." They are eminently helpful in that self-culture which so much concerns you. There is a world of worthy wisdom in Hamlet's advice to his mother,—

> "Assume a virtue, if you have it not.
> That monster, Custom, is angel yet in this:
> That to the use of actions fair and good,
> He likewise gives a frock or livery,
> That aptly is put on: Refrain *but once*,
> And that shall lend a kind of easiness
> To the next abstinence; the next more easy,
> For use can almost change the stamp of nature,
> And either curb the devil, or throw him out,
> With wondrous potency."

Connected with this matter of manner and demeanor to your patients, is the proper management of interruptions of your work while they are in the chair, the unexpected things that turn up, which modify your plans of operation, and the length of time you detain them at a sitting. In all these things, let their convenience and advantage have their honest weight with you, and let them know and see it. Especially, never let your own profit govern you to their disadvantage, and let them think so. Frankness will secure the accommodations which you ought to ask, without grudging or complaint; and remember that you have no right to do anything hastily or idly, because you have a great deal of work, or a little of something else to do. Be really true and faithful, and you will be trusted, believed, and even indulged when occasion requires it. Treat your *gratis patients* especially well. Slights to them are insults as well as injuries. Do your most generous things always in the most generous manner. A grudge will grow out of a favor very naturally, when it is really mixed up with it by yourself. Seeming indifference, and long postponement, are likely to be unkindly construed. Be careful, therefore, of the feelings of those who are not fully remunerating patients. If you tell a rich lady that you cannot attend to her for two, three, or more weeks; or if you have an appointment with her which you wish to break, she may scold you and feel disappointed, but she will not be hurt or offended, for she never suspects a slight; but less fortunate and less flattered people will think, "if I could recompense him fully, either in money or patronage, he would not have made me wait all these weeks." When you rain grace, come down handsomely like a spring shower; never drizzle, or your generosity will be *mist.*

The dismission of your patient often brings with it more or less talk, questioning and criticising the work that you have done. Don't be too much delighted with it yourself, nor too pressing in exacting praise from the patient. He may feel that his judgment is not very good, and that he has not had time to form it fairly, and he don't like to be committed for an approving opinion in advance of a thorough trial. He *may* suspect that there is some fault to be covered, and that his judgment is to be forestalled; and as to your own delight, besides the exaggerating conceit, which shows itself so plainly, there is a correct notion against you that superficial judges are the most liable to such raptures. A self-poised, thorough-bred man is not timid, doubtful, or cautious, but he is moderate and collected, as well as firm in all the assurance he feels about his own performances, for he best knows how far from perfect the most skilful are. Just here, there is a strong temptation, also, to talk about the teeth of other patients, who may be known to your present company. Now, *if* their teeth are very good, and your work has been very successful for them, you may say so in general terms, when they are referred to; but be not too particular in description, for this will excite comparison, where there is perhaps no proper parallelism of conditions, and mis-judgments will result. Indeed, if you fail of the same facility in description, or temper praise a little lower of some other case mentioned, your judgment will be so reported, and you will hear, before long, that you have not done equal justice to one or other of your patients.

All these mischiefs and indiscretions arise out of too much talk about your practice, which is the very thing you are least obliged to proclaim. The influence of the patient that you are now gossipping with, is all that you can gain by it, and that is best secured by the fine quality of your work in his or her case. Let her show *that*, and talk about it herself, and you will have your fame authenticated better than all bragging can do. A man need *not* put his light under a bushel; let it blaze on its own candlestick in his chamber; he will cut a sorry figure when he is caught carrying it out into the broad *sunlight* to show how it shines. The man that carries his own candle about with him will be conspicuous enough, indeed; but discerning people will think he would look rather better in any other light, and perhaps that he is, after all, only standing in his own light, in another sense than he imagines.

As to the matter of fees—which, if you have not yet thought of, it is to be presumed you *will*, some time or other in the course of your practice—I will take occasion, now, to say that I very well understand the importance of the subject, and, I believe, something of the philosophy of it besides. A few general suggestions only lie fairly in the track of this discourse, however, and they must be hastily presented. The amount chargeable for our operations is so controlled by the varying standards of place, custom, quality, and condition of patients, that arithmetic must be dispensed with in the discussion; the policy and principle of the thing only are within my present reach.

The first thing I have to say is, always make your work first-rate, whatever the price you may be obliged to accept for it. This will at last bring you a due appreciation, and then large fees will be sure to follow. Don't measure your skill too nicely by its reward; work for the *future*, if the present does not fully pay, and you will gather your bread from the waters when the *tide* rises, which it will be sure to do in its season. You never can charge a liberal fee to *any* one, till he is assured of your deserving. To be *paid* for extra ability, you must have the reputation of it; work, then, in the beginning for the reward which an established reputation will bring you. It is a good investment. You never can charge by your *own* estimate of your work—it is upon your reputation that you build up your fees; lay that foundation broad and strong. It is a mistake in the laws of business for a man to say, "my best work is worth *more* than I charge for it, therefore I must bring the quality down to the price, for this is but justice to myself, and it is not unjust to others." In such a judgment, the insurance of a high reputation, the security of a warranting stamp, are quite overlooked. The public pays for the *thing*, and the *certainty* of its excellence, both. The latter, in matters of art, is a considerable part of its real price; you must have such a reputation before you can *charge* for it, and you must *earn* it before you *get* it. If you pursue the opposite course, you will never either *get a* character or *deserve* it. Sow good seed in the spring, and though it seems buried now, summer will bring it all up in the fullness of the harvest. You should, in fact, follow your profession for the love of it; the extras that you do on your own account, at first, will prove that this sentiment governs you. Some day your patients will hear an established dentist say, that such work as yours is worth more money than you have charged. He will hear such a man complain of so low a rate of fees, and then the work done in a generous devotion to your art, upon your own account, will be posted to your credit. Moreover, you want business, and abundance of it; for all reasons, reasonable rates at first will help you to this, and then the public will give you your choice of patients, and your own fair command of charges. It is the way of the world, and I do not say it is a wrong way. Little rills must run down hill, but freshets and high tides make nothing of running *up* hill. An old play puts this, pithily, and pointedly; old Mirabel, in the play of the Inconstant, asks his son whether he wants any money: "No, thank you, I have plenty," replies the artful young rogue. "Then here's some for you, my boy."

"To him that *hath* shall be given," is the rule. Manage, therefore, to have the kind of thing that you want, but by fair means, so that there shall be no awkward settlements afterwards, and your stock will grow rapidly. The effect of plenty of business results from the fact, that it signifies the confidence of a great many people, and proves your competency to those who have no other means of judging. But remember, through all just and honorable policies, that you are a member of an honorable profession, which you are to serve, and improve, and honor. While your business increases, see that your quality of work improves, at least in an equal ratio. You are advancing in fees, your time pays better; all the rewards and honors of a steady and enlightened endeavor are accumulating; you can now receive the remuneration ofyour best skill, and, in all honor and honesty, you are bound to give it.

Up with your prices, and up with your work. You owe this to the fraternity, to your science, to a liberal public, that wants neither cheap talents nor gratuitous labor, and you can afford to maintain such high position while you deserve all its successes. One of the best things about high prices and a handsome practice is, that you get rid of uncomfortable people. My own experience is, that those who are disposed to huckster about the fees are hardest to please with the work; they have good reason to fear the same spirit which they feel, and they have no proper appreciation of anything liberal in your aims, or honorable in your conduct, for the lack of the same qualities in themselves. Work for a reputation that will put you out of their reach, and them out of your way. Above all, never forget that you are in debt to those who have achieved the discoveries in your profession, and improved its practice for your accomplishment. Regard the debt to our predecessors as an entailed one, and see that you pay it all over, with interest, to those who are to succeed you. Thus, gentlemen, fees, fidelity, and fame run together. May they all abound to you.

Treating, or intending to treat, a range of subjects, embracing the duties, decencies, and dignities of the practitioner and the man, in such amplitude of detail as I have indulged, you will have noticed that I have steered clear, while I was sailing all round, the use and abuse of rum and tobacco.

I believe I have acquired some reputation as a rather rough rider of these hobbies, and I have, in fact, refrained from mounting them until I could change my dress, and trot them out by themselves. They are nags well known to be *strong-winded*, and hard in the *mouth*, and I think that a dentist ought to handle them with a specially stiff *bit*. They are an ugly pair of matches, illy broken, and worse trained, and demand so much of the jockey in their management, that it is very difficult to preserve the manner of a gentleman, through the discipline they require; and so I thought it was best to bring the brutes out by themselves, for I think they are unfit for any company but their own. The one, you know, is anything but sure-footed, and the other is addicted to a most vicious and villainous *use* of his teeth. In fact, the first is even liable to fits of the staggers; and the latter, between puffing, snuffing, and frothing at the mouth, is one of the most disagreeable of beasts. Neither of them was ever known to be clean, for nobody would touch either with anything but a whip, till they are completely broken.

My reason for not showing them up in their place and order in my discourse, is, because they are not fit for any place, and are themselves always in disorder. I do not know, from either reason or experience, in what vice, or necessity of the human constitution, the indulgence in these narcotics arises. But I *do* know, *both* by reason and experience, that they are unnecessary either to study, labor, or enjoyment; and, by the unblunted senses and sentiments of abstinence from their use, I know only too well how noxious and abominable they are. I do not here occupy the place of a lecturer upon the general evils of these various forms of intemperance, with a view to the social and moral reform of the community; but I am legitimately concerned with their offensiveness, their impropriety, and indecorum in the practitioners of our profession, and I ask you to hear the just complaint to which they are liable, and to consider your own duty in regard to them.

It is generally said that experience is the *best* teacher, but it is, nevertheless, just as true, that wrong-doers do not learn the right by it. It is an invariable law, that the practice of an evil is always accompanied by bondage to it; and whether it is a mercy or a curse to the sinner, he is always blind and insensible to his vice, in the proportion that he is enslaved.

It is a *straight* edge that detects the departures of a crooked one from the line; a habitually clean hand that feels the soil of dust, and a sober man who sees and understands the grossness of intoxication.

Rum drinkers and tobacco chewers must hear the truth about their habits from the abstemious, or they will never know it, and their incapacity for a correct judgment in the premises, of right obliges them to accept the opinion of those whose purity and sensibility qualifies them for giving true testimony. A smoker needs to be told that he smells offensively, even in an omnibus, with all the windows open; that the odor hangs upon his clothing for weeks after they are laid aside in his clothes-press; that even the new clothing sent home from the tailors, looking as fresh and pure as marriage garments, are often unendurable, from the segar-smoke that was worked into them on the journeyman's bench.

Fresh smoke of a fine segar in the open air may not be disagreeably scented to every nose; but the smoker himself is disgusted with the lees and dregs of the poisonous distilment which attach to the person and dress of those who have the habit. Extemporaneous washings and brushings in preparation for a visitor or a visit are next to nothing,, they deceive nobody but the deceiver, for the foulness is in him, and over and around him, and it is not a lick and a promise, that will serve to hide it. I say it is in him, in his blood, and flesh, and breath, his skin and hair, beyond the reach of soap and water, too deep for gargles, and too strong for the counter-stench of cologne, bay rum or any thing but assafoetida. I am assured by a gentleman who subjected himself to a steam vapor bath, five days after he discontinued chewing, that the whole room was filled with the effluvia which the vapor boiled out of him. The attendant hurried him off to the cold shower bath, and threw up the windows for his own relief. You know that the absorbents of the mouth, as well as the secreting vessels are at full play upon the quid, and you cannot doubt that the fluid extract will thence permeate the whole frame.

The fact that foreign substances enter the circulation in their formal state, and prove their presence in organs most distant from the gate they enter at, by the display of all their effects there, is too well proved to admit of question. If you take sulphur, it will color a silver watch in your pocket. Arsenic used for destroying the nerve of a tooth, must be carefully watched and guarded, for, if neglected, it will sometimes find its way from the cavity into the system, and work its mischief as certainly as if taken into the stomach. The garlic eaten by a cow, is strong upon her butter, new clover tastes her milk. Madder colors the *bones* of young animals fed upon it, and rhubarb taken by a nurse, can be detected in the alvine dejections of the child. If digestion, circulation, and all the sifting and straining that a substance encounters in its passage through the body, thus leaves it so little changed, especially in the case of aromatics, poisons and medicines, it is easy to believe, that every fibre and organ of the body, may be penetrated by that substance which in its way is all these put together.

The same thing is true of all the kinds of stimulating liquor. Dr. John Hunter and his pupils, affirm that they saw the blue flame, and detected the peculiar odor of burning alcohol in the brain of a drunkard, which they subjected to the experiment, for the purpose of ascertaining the fact of such absorption.

The Professor of Physiology, has in the course of his lectures, probably had occasion to speak of the function of pulmonary transpiration. You are well informed, and well assured by other evidence which corroborates your scientific teachings, even by the authority of your own senses, that the lungs are among the principal emunctories of the system. Their service as excretory organs, is in fact not behind that of the skin and kidneys themselves. Scarce a scent or odor of any article of drink or diet, but is rendered back again by this route, which was before admitted through the mouth into the stomach. The breath comes up as from a sink loaded with the pestilence, imbibed in gross indulgence. Who has not smelled the *double distilled* fumes of whiskey, all the worse for the *last* process, of ale, onions, oysters, garlick and tobacco upon the breath. What scavenger work these lungs of ours have to do! and sorry tell-tales they are too. They are vocal as well as respiratory organs you know, but their *loudest* utterings are nothing to the whispers low but deep, which they give out, of our habits regular and irregular. Shakespeare who misses nothing, has hit it finely, and deservedly placed it among the horrors that Cleopatra apprehended, when she should be led captive through the streets of Rome.

> "Now Iras what thinkest thou?
> Thou, an Egyptian puppet, shall be shorne
> In Rome as well as I: Mechanic slaves
> With greasy aprons, tools and hammers, shall
> Uplift us to the view—In their *thick breaths*,
> *Rank* of gross diet, shall he be enclouded,
> And forced to drink their vapor."

I could'nt go wrong in such a matter by following my own nose, if it is proverbially a good guide in other things, it is quite infallible in this, and I am not without authority you see, the authority of our own books, of science, and that most wonderful of all directories, in thought and conduct, that richest treasury of truth, speculative, and practical, that revelation of human nature, in all its heights and depths, the oracular Shakespeare. But if from these, there are any so hardy, or so obstinate as to take an appeal, the uncorrupted senses of every cleanly man shall be our umpire. Now please to figure your position at the operating chair. Your patient a delicately decent woman, with her mouth held open as wide as the lips permit, her nerves excited to their utmost keenness, and the operator hanging over her upturned face. A back tooth is to be seen and reached, a constrained position to be maintained by the operator, his face directly opposed, and as near as possible, and directly in the line of the open throat and nostrils, pumping at every stroke of his lungs the fetid breath of a rum sink or a tobacco mash, like a gasometer into her revolted senses! I say it is hideous, I have no fitting words which it were fit to use in the description of the outrage.

As a question of conduct in professional practice, as a *condition* of success in *that* practice, the lightest word to be used about it is too heavy for one gentleman to throw directly at another. Therefore, again, I protest that I am not personal. Indeed, I am most happy to know, that while the medical colleges here and elsewhere, are infamous for these abuses; their lecture halls horrible with the stench of stale tobacco smoke, and the floors all covered with maps of the great lakes drawn in tobacco spit, rendering them tenfold more horrible than the dissecting rooms themselves in the warm dampness of the first days of March, our rooms are clear of the offense.

Many thanks, gentlemen, most sincerely and earnestly tendered to you for this among the many other pleasures I have found in our intercourse through this session.

I am convinced, and you will soon learn, that this matter has all the importance that I give it. I am fully convinced that it is not in caprice, or passion, pride or prejudice, nor is it in the compass of the English language, to exaggerate the abominations which I am feebly endeavoring to expose.

You will have perceived that in these two lectures devoted to professional conduct, and office economy and order, as in other instances of my prelections, I have had an eye, as occasion offered, to things which concern our profession, its honor, rank and general character, as well as to the strictly technical duties of my office. For my life I cannot sink the *man* and the profession, in the *trade*, nor even in the science which we pursue. Nor can you, without sinking the man, profession, trade and science, all together. Hence, as the Latin adage has it: "Hinc illae lachrymae," "hence these tears," and whatever of railing and railery has gone along with them. And while I cannot wholly over pass the reflex influence that the decorum and justice of professional conduct exert upon the man and the gentleman I am not without the impulse, also, to say a timely word for the stand my brethren ought to take, and have so much power to make effectual toward the amelioration of the communities in which they must hold a conspicuous position. The honors and confidence which the public gives us, will be cheapened sadly, if we do not wear them usefully.

So far as beneficent reforms *rest* upon *reasons* that are to be found in our science, so far as they can be properly affected by our example, it is a debt of conscience to perform the social duty. We must not allow our own conduct to be construed against the truth of our own art. That from which the laws of health with which we are conversant should dissuade, must not have the *false warrant* of our *example.* That would be bearing false witness against our neighbor.

Ninety-six millions of gallons of ale and spirituous liquors were produced in the United States in 1852, and three hundred millions of barrels, or one hundred thousand tons of tobacco were grown in the same year—so says the census. If all this intoxicating liquor and more, were consumed, and half the quantity of the weed, by our fellow citizens, which is a fair estimate; it is allowable in the well wishers of the race, to raise their voices against these monstrous evils, and ours ought not to be wanting, as citizens, philanthropists, and especially as guardians in our proper sphere, of the public health. The report shows that the people of this country pay as much for imported segars, as they receive for exported wheat, and drink in the form of French brandy the whole proceeds of the Indian corn exportation.

These high figures are truly astounding. I see not how we of all men, can look at them without concern—and may I not say to you in all confidence of their deserving your deepest consideration. "Think of these things," and as this phrase reminds me of its author, allow me to quote that fine summary of St. Paul to the Phillippians, with which he concludes so many noble and excellent advices.

"Finally, brethren, whatsoever things are true, whatsoever things are venerable, whatsoever things are just, whatsoever things are pure, whatsoever things are lovely, whatsoever things are of good report, if there be *any* virtue, and if there be any praise—*Think of these things*."

